# Up‐regulated CST5 inhibits bone resorption and activation of osteoclasts in rat models of osteoporosis via suppression of the NF‐κB pathway

**DOI:** 10.1111/jcmm.14552

**Published:** 2019-08-11

**Authors:** Fei Wang, Chuanzhu Zhang, Wei Ge, Guoqiang Zhang

**Affiliations:** ^1^ Department of Pain Linyi People's Hospital Linyi China; ^2^ Department of Anesthesiology Linyi People's Hospital Linyi China; ^3^ Department of Orthopedics Chinese Medicine Hospital in Linyi City Linyi China; ^4^ Department of Hand and Foot Surgery Linyi People's Hospital Linyi China

**Keywords:** activation of osteoclasts, bone resorption, CST5, NF‐κB pathway, osteoporosis

## Abstract

Here, we aim at exploring the effect of CST5 on bone resorption and activation of osteoclasts in osteoporosis (OP) rats through the NF‐κB pathway. Microarray analysis was used to screen the OP‐related differentially expressed genes. Osteoporosis was induced in rats by intragastric retinoic acid administration. The serum levels of tartrate‐resistant acid phosphatase (TRAP), bone alkaline phosphatase (BALP) and osteocalcin (OC) and the expression of CD61 on the surface of osteoclasts were examined. The number of osteoclasts and the number and area of resorption pits were detected. Besides, the pathological changes and bone mineral density in bone tissues of rats were assessed. Also, the relationship between CST5 and the NF‐κB pathway was identified through determining the expression of CST5, RANKL, RANK, OPG, p65 and IKB. Poorly expressed CST5 was indicated to affect the OP. CST5 elevation and inhibition of the NF‐κB pathway decreased serum levels of TRAP, BALP and OC and expression of CD61 in vivo and in vitro. In OP rats, CST5 overexpression increased trabecular bones and bone mineral density of bone tissues, but decreased trabecular separation, fat within the bone marrow cavities and the number of osteoclasts through inhibiting the NF‐κB pathway. In vivo experiments showed that CST5 elevation inhibited growth in number and area of osteoclastic resorption pits and restrained osteoclastic bone absorption by inhibiting the NF‐κB pathway. In summary, overexpression of CST5 suppresses the activation and bone resorption of osteoclasts by inhibiting the activation of the NF‐κB pathway.

## 1 INTRODUCTION

Osteoporosis (OP) is a kind of metabolic bone disease with features of decreased bone mass, increased incidence of fracture and altered bone architecture.^1^, ^2^ OP is considered to be the outcome of losing balance between osteoblasts which take responsibility for bone formation and osteoclasts which for bone resorption.^3^ The elders are more likely to suffer from OP, and it plagues women much more than men.^4^ As bone endures complicated remodelling frequently, old bones will be replaced to keep the skeleton in a better state.^2^ Still, many osteoporotic sufferers have not received appropriate treatment, which is partially due to patients’ worry concerning drug safety.^5^ Therefore, finding new drugs with better effect and less adverse effects for OP treatment is actually an urgent requirement.

As a suppressor of lysosomal and secreted cysteine proteases, cystatin D (CST5) degrades multiple targets which contain matrix components, adhesion proteins and other proteases.^6^ Cystatin D is a member of cystatin family II, which is from the cystatin superfamily.^7^ It was demonstrated that CST5 expressed lowly in patients with bladder cancer and human immunodeficiency virus type 1 (HIV‐1).^8^, ^9^ Also, a previous study has proved that CST5 can inhibit xenograft tumour growth in vivo through restraining the Wnt/β‐catenin pathway.^10^ Interestingly, CST5, encoding cystatin D, is induced by the vitamin D receptor (VDR) directly, which is reported to work as a tumour suppressor.^11^ Located in tandem on chromosome 20 (20p11.21) in human, CST5 is involved in the inhibition of cysteine peptidases and antimicrobial activities.^12^ Additionally, the down‐regulation of receptor activator of nuclear factor (NF)‐κB ligand (RANKL) was previously clarified to depress osteoclast formation, bone resorption and attenuate original OP.^13^ RANKL can stimulate RANK, activate the NF‐κB pathway and then contribute to osteoclast differentiation, activation and survival.^14^ Furthermore, it was revealed that inhibition of the RANKL pathway led to inhibited formation and activity of osteoclasts.^15^ More importantly, the NF‐κB pathway was previously reported to be regulated by cystatin E/M suppressor gene, in which reduced cystatin E/M could reduce the expression of the NF‐κB pathway‐related factors.^16^ All the above data led us to a hypothesis that CST5 might act as a potential therapeutic target for OP treatment through regulating the NF‐κB pathway. Then based on this hypothesis, we conducted a series of experiments to prove the relationship between CST5 and NF‐κB. Both in vitro and in vivo experiments displayed that the alteration of CST5 expression triggered the change of the NF‐κB pathway. Besides, the addition of NF‐κB pathway inhibitor BAY11‐7085 had the same effects with CST5 overexpression, which further determined the relationship between CST5 and NF‐κB pathway and further proved our hypothesis.

## 2 MATERIALS AND METHODS

### 2.1 Ethics statement

The animal experiments were conducted in strict accordance with the Guide for the Care and Use of Laboratory Animals of the National Institutes of Health. The experiment protocol was approved by the Animal Ethics Committee of Linyi People's Hospital. All efforts were made to minimize suffering of animals.

### 2.2 Microarray analysis

The OP‐related gene expression data set GSE63009 was obtained after retrieving the Gene Expression Omnibus (GEO) database (https://www.ncbi.nlm.nih.gov/geo/) with “osteoporosis” as the keyword. The GSE63009 gene expression data set included the expression data of alendronate‐ or risedronate‐treated osteoclasts and control osteoclasts, and the chip was annotated by GPL570‐[HG‐U133_Plus_2] Affymetrix Human Genome U133 Plus 2.0 Array. The limma package^17^ of R language was applied for standardization of expression matrix and screening of differential expressed genes (DEGs), and then the heat map of DEGs was drawn. We regarded |log fold change (FC)| > 2 and *P* < .05 as the screening criteria for DEGs.

### 2.3 Establishment of rat models of OP

Totally, 80 healthy male Sprague Dawley (SD) rats aged 6‐7 months with a weight of 230‐330 g (purchased from the animal experiment centre in Linyi People's Hospital) were used in this study and fed with standard diet. Rats were randomly grouped into 2 groups: 10 rats were used in normal group, and the other 70 rats were used for establishing the model of rats with OP by intragastric administration of 100 mg/kg/d retinoic acid.^18^ Then, 60 successfully modelled rats with OP were randomly assigned into OP group (without any treatment), shRNA‐CST5 group (each rat was intraperitoneally injected with 5 × 10^11^ vector genomes per gram shRNA‐CST5 per day), CST5‐overexpression (OE) group (each rat was intraperitoneally injected with 5 × 10^11^ vector genomes per gram overexpress‐CST5 per day), BAY11‐7085 group (each rat was intraperitoneally injected with 20 mg/kg NF‐κB pathway inhibitor BAY11‐7085 per day), shRNA‐CST5 + BAY11‐7085 group (each rat was intraperitoneally injected with 200 μL shRNA‐CST5 and NF‐κB pathway inhibitor BAY11‐7085 per day) and negative control (NC) group (each rat was intraperitoneally injected with 200 μL normal saline per day).

### 2.4 Micro‐computed tomography (micro‐CT) assessment

Live rats were subjected to the micro‐CT analyses under anaesthesia before being euthanized at the end of this study. Micro‐CT imaging of joints was performed using a Siemens Inveon CT/PET Multimodality system.^19^

### 2.5 Haematoxylin and eosin (HE) staining

The muscles and connective tissues were stripped from the thigh bones of rats and then soaked in 10% formaldehyde solution for 3 days. After being washed with phosphate‐buffered saline (PBS), tissues were decalcified with 10% ethylenediaminetetraacetic acid (EDTA) for 4 weeks. Tissues were then routinely dehydrated, cleared, waxed, embedded and cut into 10 μm decalcified sections. After dewaxing and hydration, sections were stained by haematoxylin for 5 minutes, rinsed in tap water for 1 minute, differentiated by 1% hydrochloric acid ethanol for 30 seconds, soaked in tap water for 15 minutes, stained with 0.5% eosin for 3 minutes and washed with distilled water. Lastly, sections were dehydrated, cleared and sealed with neutral balsam, and the pathological characteristics were observed under an optical microscope.

### 2.6 Enzyme‐linked immunosorbent assay (ELISA)

The experimental procedure was conducted according to the instructions of the ELISA kits of tartrate‐resistant acid phosphatase (TRAP; Life Diagnostics), bone alkaline phosphatase (BALP; R&D Systems) and osteocalcin (OC; NeoScientific). The rat serum samples were added into a 96‐well plate to combine with the capture antibody on the plate bottom and incubated for 1 hour at room temperature. Samples were washed before adding with enzyme‐labelled antibody (ELA) and incubated for 30 minutes. The plate was washed three times and added with 3,3′,5,5′‐tetramethylbenzidine (TMB) for developing. Reaction was stopped by adding with sulphuric acid. The optical density (OD) value was read at 490 nm using a microplate reader, and the concentrations of TRAP, BALP and OC were calculated in accordance with the OD value.

### 2.7 TRAP staining

Staining solution was prepared according to the instructions of TRAP staining kit (Sigma). Paraffin‐embedded sections were dewaxed and hydrated, soaked in preheated dye liquor at 37°C, washed with distilled water and stained again for 1 minute. Then, the sections were dehydrated with gradient ethanol, cleared with dimethylbenzene and sealed with neutral balsam. Microscopic images were obtained, and then, the number of osteoclasts per bone surface (N.Oc/BS) was analysed using Image‐Pro plus analysis software (Media Cybernetics). The nucleus was stained blue. The positive expression of TRAP was expressed as bright or deep red granules occurred in the cytoplasm. All the giant cells with TRAR‐positive expression containing 3 or more nucleus were considered as osteoclasts.

### 2.8 Osteoclast treatment and grouping

Two 4‐week‐old SD rats were sacrificed by cervical dislocation method, and the lumbar spines L_1 − 5_ of rats were separated under aseptic conditions. Soaked in 75% ethanol for 3 minutes, lumbar spines were rinsed using Hank's balanced salt solution (BSS), and then, the bone tissues were cut into pieces and centrifuged using α‐minimum essential medium (α‐MEM) (10 mL). The cell suspension was collected, filtrated by cell strainer and added with 10 mL α‐MEM again to centrifuge. After three repetitions, cell suspension was centrifuged with the supernatant discarded. The precipitate was added with osteoclast inducing medium and then added into a 24‐well plate with prepared bovine cortical bone slices.^20^ Cell suspension was then incubated with 5% CO_2_ at 37°C for 24 hours and then incubated in the osteoclast inducing medium once. Osteoclasts were identified by TRAP staining and then grouped into shRNA‐CST5 group (each well was added with 2.0 μg shRNA‐CST5 plasmids), OE‐CST5 group (each well was added with 2.0 μg OE‐CST5 plasmids), BAY11‐7085 group (each well was added with 20 μL NF‐κB pathway inhibitor BAY11‐7085 plasmids), shRNA‐CST5 + BAY11‐7085 group (each well was added with 2.0 μg shRNA‐CST5 and 20 μL BAY11‐7085 plasmids), NC group (each well was added with 2.0 μg empty vector plasmids) and blank group (without any treatment).

### 2.9 Flow cytometry

On the 7th day after different treatments, cells of each group were detached by trypsin, centrifuged and then collected. Cell density was adjusted to 1 × l0^6^ cells/mL by PBS. A total of 100 μL cells were added with 1 μL CD61‐fluorescein isothiocyanate (FITC) monoclonal antibody, mixed, incubated avoiding exposure to light for 20 minutes, added with 500 μL PBS and then examined using flow cytometry. The instrument was adjusted using standard fluorescent beads to keep coefficient of variation lower than 2%. Ten thousand cells were collected after being cultured for 7 days, and the data were analysed by Expo32 for windows software (Beckman Coulter). Expression was presented as the mean fluorescence intensity.

### 2.10 Toluidine blue (TB) staining

Ground sections of bone cultured in the plate were taken out after 7‐day culture. Sections were fixed with 2.5% glutaraldehyde for 10 minutes, ultrasonically cleaned by 0.25 mol/L ammonium hydroxide 3 times, dehydrated by gradient ethanol and dried out. Sections were stained using 1% TB, washed with distilled water and dried. Six fields in each section were selected randomly under an optical microscope, and the resorption pits were presented blue in elliptical shape or irregular shape. The number of resorption pits was observed and recorded to obtain the mean value as the result. Area of resorption pits was analysed using the Image‐Pro Plus Analysis software (Media Cybernetics) to reflect the bone resorption activity of osteoclast with the pixel as the unit.

### 2.11 Reverse transcription‐quantitative polymerase chain reaction (RT‐qPCR)

Bone tissues or cells in the logarithmic growth phase were collected. The total RNA was extracted using the TRIzol method (Invitrogen) and reserved at −80°C. Total RNA was reversely transcribed into complementary DNA (cDNA) using PrimeScript@RT reagent kit (Perfect Real Time; TaKaRa) and reserved at −20°C for subsequent experiments. RT‐qPCR was performed using ABI7500 quantitative PCR system (ABI) with β‐actin as an internal reference. Reaction conditions were as follows: pre‐denaturation at 95°C for 5 minutes, 40 cycles of denaturation at 90°C for 30 seconds, annealing at 60°C for 40 seconds and extension at 72°C for 40 seconds. The primer sequences are shown in Table 1. Each sample was repeatedly measured 3 times. PCR results were analysed using Opticon Monitor 3 software (Bio‐Rad). Each sample was repeatedly measured 3 times. The relative expression of the ratio of related genes to internal reference β‐actin was calculated using the 2^−ΔΔCt^ method, and then, the statistical results were obtained.

**Table 1:** Primers used for RT‐qPCR analysis

Gene	Primer sequence
CST5	F: 5′‐TGCTGCTGACTGCCTTGATG‐3′
	R: 5′‐CACACTGCACACTCTTGTCAT‐3′
p65	F: 5′‐TGGCAGTGATTCACGAAGCC‐3′
	R: 5′‐CTGTGCCTAGCAGAGGGTT‐3′
IκBα	F: 5′‐CTGGTCTCGCTCCTGTTGA‐3′
	R: 5′‐GCCCTGGTAGGTTACTCTGTTG‐3′
RANK	F: 5′‐TTGCCCGGTTTAATAATTCTGCTTCTCTTC‐3′
	R: 5′‐TGTATTCATCTTCTGTGGGCATCTGTCTG‐3′
RANKL	F: 5′‐AGCGTCGCCCTGTTCTTCTATTT‐3′
	R: 5′‐GCCATCCACCATCGCTTTCTCTG‐3′
OPG	F: 5′‐TCCTGGCACCTACCTAAAACAGCA‐3′
	R: 5′‐CTACACTCTCGGCATTCACTTTGG‐3′
β‐actin	F: 5′‐CTGACACCTTCACCATTCCAG‐3′
	R: 5′‐ATTGCTGACAGGATGCAGAAG‐3′

Abbreviations: CST5, cystatin D; F, forward; OPG, osteoprotegerin; R, reverse; RANK, receptor activator of nuclear factor‐κB; RANKL, receptor activator of nuclear factor‐κB ligand; RT‐qPCR, reverse transcription‐quantitative polymerase chain reaction.

### 2.12 Western blot analysis

Protein lysis buffer (Beyotime Biotechnology Co., Ltd.) was added into bone tissues or cells in the logarithmic growth phase to extract the total protein. Protein quantitation was conducted using the Bradford method (Thermo Fisher Scientific Inc). Next, 50 μg total RNA was subjected to 12% sodium dodecyl sulphate‐polyacrylamide gel electrophoresis (SDS‐PAGE) and then transferred onto a polyvinylidene fluoride (PVDF) membrane (Millipore). The membrane was blocked with 5% skim milk at 37°C for 1 hour and then incubated with the rabbit anti‐rat monoclonal antibodies CST5 (MAB1202, Bio‐Techne China Co., Ltd.), p65 (NB100‐2176, Bio‐Techne China Co., Ltd.), IKB (NBP2‐23901, Bio‐Techne China Co., Ltd.), receptor activator of nuclear factor‐κB (RANK; ab200369, Abcam Inc), receptor activator of nuclear factor‐κB ligand (RANKL; ab9957, Abcam Inc), osteoprotegerin (OPG; ab73400, Abcam Inc) and β‐actin (ab8226, Abcam Inc) at 4°C overnight. The membrane was rinsed 3 times (5 minutes for each) using phosphate‐buffered saline Tween 20 (PBST) and added with horseradish peroxidase‐labelled goat anti‐rabbit secondary antibody (1:4000, Cell Signaling Technology), followed by incubation at room temperature for 2 hour. Chemiluminescent liquid was prepared by mixing the Luminol Reagent and Peroxide Solution (Millipore) at the ratio of 1:1. The membrane was scanned, photographed and analysed. The relative expression of protein was presented as the ratio of grey value of target band to that of internal reference band. Each experiment was repeated 3 times, and the mean value was calculated.

### 2.13 Statistical analysis

Statistical analysis was conducted using SPSS 21.0 (IBM Corp. Armonk), and measurement data were expressed as mean ± standard deviation (SD). Multiple groups were compared by one‐way analysis of variance (ANOVA). The least significant difference (LSD) test was used for pairwise comparisons. *P* < .05 was considered significant different.

## 3 RESULTS

### 3.1 CST5 is speculated to affect OP through mediation of the NF‐κB pathway

**Figure 1:**
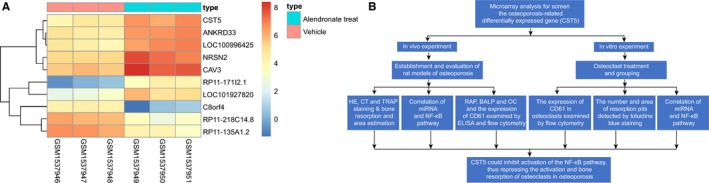
CST5 might affect OP progression by regulating the NF‐κB pathway. A, the heat map of top 10 differentially expressed genes related to OP screened from the GSE63009 gene expression data set. The abscissa indicates the sample number, and the ordinate indicates the differentially expressed genes. The histogram in the upper right is the colour gradation. Each rectangle in the figure corresponds to a sample expression value. Red indicates high expression level, and blue indicates low expression level. B, the flow chart of this study. OP, Osteoporosis; CST5, Cystatin D

The GSE63009 gene expression data set was selected from the GEO database. With the untreated osteoclasts used as the control group, the gene expression changes of alendronate‐treated osteoclasts were analysed. A total of 86 DEGs were obtained according to the screening criteria *p* value < .05 and |logFC| > 2. The heat map of the top 10 DEGs was drawn. As shown in Figure 1A, the expression of CST5 significantly increased in alendronate‐treated osteoclasts, indicating that poorly expressed CST5 might influence the osteoclast function. CST5, a cysteine protease inhibitor, has been reported to be associated with tumour suppression,^10^, ^11^ but there was little research focusing on the effect of CST5 on OP. The role of another cysteine protease inhibitor, CST3, was reported to reduce the formation of osteoclasts by interfering with the late differentiation of osteoclast precursors.^21^ Studies have shown that inhibition of the NF‐κB pathway could relieve OP.^22^, ^23^ Moreover, cystatin was demonstrated to affect the NF‐κB pathway.^16^ Combined with DEG screening results, we put forward a hypothesis that CST5 could mediate the NF‐κB pathway, thus affecting osteoclasts function and finally affecting OP. The flow chart is shown in Figure 1B.

### 3.2 Up‐regulation of CST5 and inhibition of the NF‐κB pathway alleviate pathological changes and improve bone mineral density of bone tissues of rats with OP

**Figure 2:**
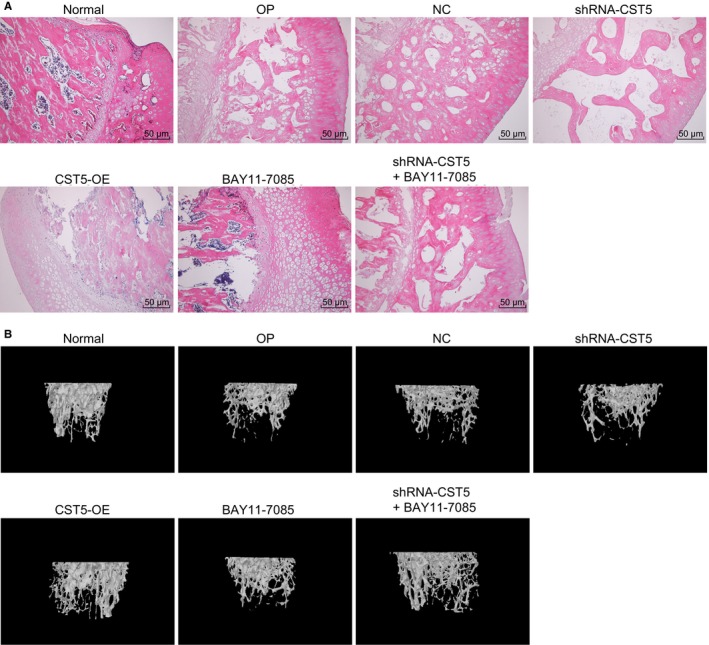
CST5 elevation and inhibition of the NF‐κB pathway contribute to relieved pathological changes and improved bone mineral density of bone tissues of rats with OP. A, HE staining (×200) for vertebral trabecular bone showed increased trabecular bone and decreased trabecular separation and fat within the bone marrow cavities after inhibition of the NF‐κB pathway mediated by CST5. B, micro‐CT assessment on bone tissues of rats with OP after inhibition of the NF‐κB pathway mediated by CST5. CST5, cystatin D; HE, haematoxylin‐eosin; NC, negative control; NF‐κB, nuclear factor‐κB; OP, osteoporosis

Haematoxylin‐eosin staining (Figure 2A) was used to observe the pathological changes in bone tissues of rats. After HE staining, the trabecular bones of rats were stained deep red, the cytoplasm in rat bone marrow stromal cells was stained yellow and nucleus were stained black. Compared with the normal group, rats in other groups showed decreased trabecular bones, increased trabecular separation and increased fat within the bone marrow cavities. Compared with the OP group, rats in the shRNA‐CST5 group showed decreased trabecular bones, increased trabecular separation and increased fat within the bone marrow cavities while the BAY11‐7085 and OE‐CST5 groups showed an opposite trend. The NC and shRNA‐CST5 + BAY11‐7085 groups showed no significant difference. Meanwhile, according to micro‐CT assessment, relative to OP group, the shRNA‐CST5 group showed lower bone mineral density, while no significant difference regarding bone mineral density was found in the BAY11‐7085 and CST5‐OE groups relative to the NC group, and no obvious change was observed between the NC and shRNA‐CST5 + BAY11‐7085 groups (Figure 2B). All the above results demonstrated that up‐regulation of CST5 and inhibition of NF‐κB pathway could alleviate the pathological changes and enhance bone mineral density of rats with OP.

### 3.3 CST5 reduces serum levels of TRAP, BALP and OC through inhibiting NF‐κB pathway in OP rats

**Figure 3:**
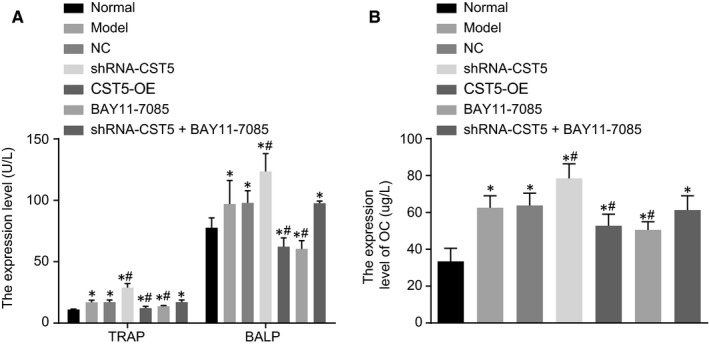
Serum levels of TRAP, BALP and OC are inhibited by CST5‐mediated the NF‐κB pathway inhibition in OP rats. A, the serum levels of TRAP and BALP in each group, as detected by ELISA; B, the serum levels of OC in each group, as detected by ELISA; *, *P* < .05 vs the normal group; #, *P* < .05 vs the OP group; n = 10; the experimental data were expressed as mean ± SD; the one‐way ANOVA was used for statistical analysis; the samples were collected from serum of rat. ANOVA, analysis of variance; BALP, bone alkaline phosphatase; CST5, cystatin D; ELISA, enzyme‐linked immunosorbent assay; NC, negative control; NF‐κB, nuclear factor‐κB; OC, osteocalcin; OP, osteoporosis; TRAP, tartrate‐resistant acid phosphatase

Serum levels of TRAP, BALP and OC in rats were examined using ELISA, and the results (Figure 3) showed that, compared with the normal group, serum levels of TRAP, BALP and OC in other groups were increased (*P* < .05). Compared with the OP group, the shRNA‐CST5 group showed higher serum levels of TRAP, BALP and OC while the BAY11‐7085 and OE‐CST5 groups showed lower serum levels of TRAP, BALP and OC (all *P* < .05). The NC and shRNA‐CST5 + BAY11‐7085 groups showed no significant difference (*P* > .05). These results indicated that the serum levels of TRAP, BALP and OC could be decreased by CST5‐mediated inhibition of the NF‐κB pathway.

### 3.4 CST5 affects the progression of OP via inhibition of the NF‐κB pathway

**Figure 4:**
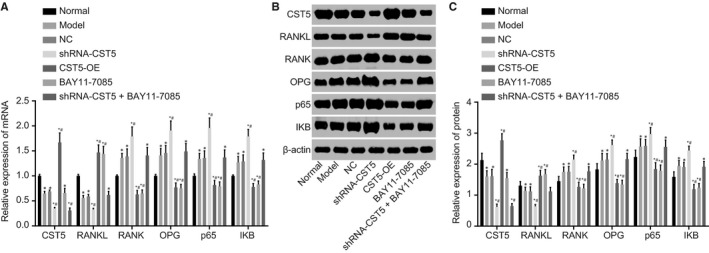
CST5 inhibits the NF‐κB pathway in OP rats. A, mRNA expression of CST5, RANKL, RANK, OPG, p65 and IKB in each group evaluated by RT‐qPCR; B, the protein bands of CST5, RANKL, RANK, OPG, p65, IKB and β‐actin in each group; C, protein level of CST5, RANKL, RANK, OPG, p65 and IKB in each group assessed by Western blot analysis; *, *P* < .05 vs the normal group; #, *P* < .05 vs the OP group; n = 10; the experimental data were expressed as mean ± SD; the one‐way ANOVA was applied for statistical analysis; the samples were collected from femoral tissues of rats. ANOVA, analysis of variance; CST5, cystatin D; NC, negative control; NF‐κB, nuclear factor‐κB; OP, osteoporosis; OPG, osteoprotegerin; RANK, receptor activator of nuclear factor‐κB; RANKL, receptor activator of nuclear factor‐κB ligand; RT‐qPCR, reverse transcription‐quantitative polymerase chain reaction

The mRNA expression and protein level of CST5, RANKL, RANK, OPG, p65, IKB and β‐actin were examined using RT‐qPCR and Western blot analysis. Results (Figure 4) demonstrated that compared with the normal group, mRNA expression and protein level of CST5 and RANKL were lower while those of RNAK, OPG, p65 and IKB was higher in the OP group (all *P* < .05). The shRNA‐CST5 group showed further reduced mRNA expression and protein level of CST5 and RANKL but further increased expression of RANK, OPG, p65 and IKB, as compared with the OP group (all *P* < .05). The BAY11‐7085 and the OE‐CST5 groups showed elevated mRNA expression and protein level of CST5 and RANKL and reduced expression of RANK, OPG, p65 and IKB in comparison with the OP group (all *P* < .05), indicating the similar effects of CST5 overexpression and inhibition of NF‐κB pathway on OP progression. The NC, shRNA‐CST5 + BAY11‐7085 and OP groups showed no significant difference (except CST5; *P* > .05). These findings provided evidence that the progression of OP could be influenced through inhibition of the NF‐κB pathway meditated by CST5.

### 3.5 CST5 suppresses osteoclast formation through the inhibition of the NF‐κB pathway

**Figure 5:**
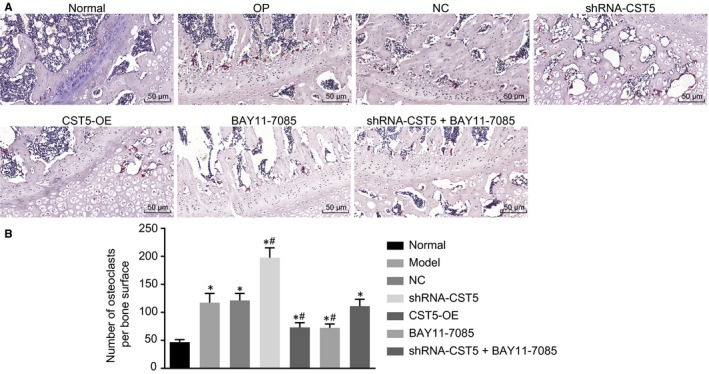
Up‐regulation of CST5 and inhibition of the NF‐κB pathway reduce the number and activity of osteoclasts in OP rats. A, the results of TRAP staining (×200) in rat femurs; B, statistical analysis of number of osteoclasts per bone surface; *, *P* < .05 vs the normal group; #,* P* < .05 vs the OP group; n = 10; the experimental data were expressed as mean ± SD; the one‐way ANOVA was applied for statistical analysis; CST5, the samples were collected from femoral tissues of rats. ANOVA, analysis of variance; CST5, cystatin D; NC, negative control; NF‐κB, nuclear factor‐κB; OP, osteoporosis; TRAP, tartrate‐resistant acid phosphatase

Osteoclasts can be stained pink with TRAP staining and deeper pink staining represented greater activity of osteoclasts. TRAP staining was used to examine the number and activity of osteoclast in the defect area of rats. Results (Figure 5) clarified that compared with the normal group, the osteoclasts in other groups were significantly increased. Compared with the OP group, the shRNA‐CST5 group showed increased osteoclasts while osteoclasts in the BAY11‐7085 and OE‐CST5 groups were decreased (*P* < .05). The NC and shRNA‐CST5 + BAY11‐7085 groups showed no significant difference (*P* > .05). All the above results suggested that CST5 could confer an inhibitory effect on osteoclast formation by inhibiting the NF‐κB pathway.

### 3.6 CST5 hinders CD61 induction by inhibiting the NF‐κB pathway

**Figure 6:**
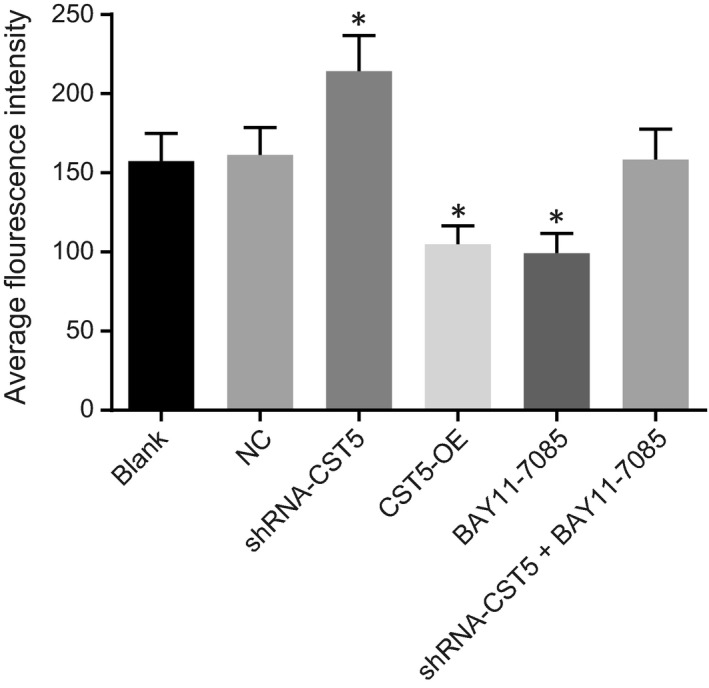
The expression of CD61 on the surface of osteoclasts is inhibited by CST5 mediated the NF‐κB pathway inhibition in OP rats. *, *P* < .05 vs the blank group; n = 10; the experimental data were expressed as mean ± SD; the one‐way ANOVA was applied for statistical analysis; the samples were collected from osteoclast of rats. ANOVA, analysis of variance; CST5, cystatin D; NC, negative control; NF‐κB, nuclear factor‐κB; OP, osteoporosis

Flow cytometry was used to examine the expression of CD61 in osteoclasts (Figure 6). Compared with the blank group, the expression of CD61 increased in the shRNA‐CTS5 group but decreased in the BAY11‐7085 and the OE‐CST5 groups (*P* < .05). The NC and shRNA‐CST5 + BAY11‐7085 groups showed no significant difference (*P* > .05). All the above results concluded that CST5 could suppress the induction of CD61 through inhibition of the NF‐κB pathway.

### 3.7 CST5 restrains growth in number and area of osteoclastic resorption pits through inhibiting the NF‐κB pathway

Toluidine blue staining was used to measure the number and area of bone resorption pits. Results in Table 2 explained that the number and area of resorption pits were increased in the shRNA‐CST5 group while reduced in the BAY11‐7085 and the OE‐CST5 groups in comparison with the blank group (*P* < .05). The NC and shRNA‐CST5 + BAY11‐7085 groups showed no significant difference (*P* > .05). To sum up, the number and area of osteoclastic resorption pits could be reduced through inhibition of the NF‐κB pathway mediated by CST5.

**Table 2:** The number and area of bone resorption pits

Group	Numbers (in each section)	Area (pix)
Blank	43.00 ± 4.58	11857 ± 831
NC	43.67 ± 4.51	12121 ± 901
shRNA‐CST5	68.33 ± 6.66*	19212 ± 1216*
OE‐CST5	29.67 ± 2.08*	8793 ± 731*
BAY11‐7085	29.67 ± 2.08*	8793 ± 731*
shRNA‐CST5 + BAY11‐7085	42.33 ± 4.16	12101 ± 895

Note

*, *P* < .05 compared with the blank group; n = 10; the experimental data were expressed as mean ± SD; the one‐way ANOVA was used to analyse data; TB staining, toluidine blue staining; NC, negative control; CST5, cystatin D.

### 3.8 CST5 suppresses osteoclastic bone absorption through inhibition of the NF‐κB pathway

**Figure 7:**
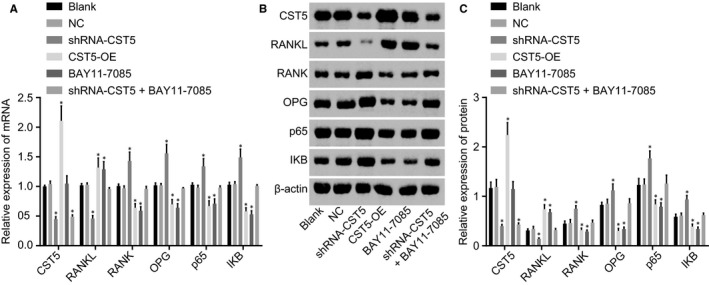
CST5 suppresses the NF‐κB pathway to inhibit osteoclastic bone absorption in OP rats. A, mRNA expression of CST5, RANKL, RANK, OPG, p65 and IKB determined by RT‐qPCR; B, the grey values of CST5, RANKL, RANK, OPG, p65, IKB and β‐actin protein bands; C, protein level of CST5, RANKL, RANK, OPG, p65 and IKB measured by Western blot analysis; *, *P* < .05 vs the blank group; n = 10; the experimental data were expressed as mean ± SD; the one‐way ANOVA was performed for statistical analysis; the samples were collected from osteoclast of rats. ANOVA, analysis of variance; CST5, cystatin D; NC, negative control; NF‐κB, nuclear factor‐κB; OP, osteoporosis; OPG, osteoprotegerin; RANK, receptor activator of nuclear factor‐κB; RANKL, receptor activator of nuclear factor‐κB ligand; RT‐qPCR, reverse transcription‐quantitative polymerase chain reaction

The mRNA expression and protein level of CST5, RANKL, RANK, OPG, p65, IKB and β‐actin were examined using RT‐qPCR and Western blot analysis. Results (Figure 7) displayed that compared with the blank group, the shRNA‐CST5 group showed decreased mRNA expression and protein level of CST5 and RANKL and increased RANK, OPG, p65 and IKB while the BAY11‐7085 and the OE‐CST5 groups showed an opposite trend (all *P* < .05). The mRNA expression and protein level of CST5 in the shRNA‐CST5 + BAY11‐7085 group decreased (*P* < .05), and no significant difference was revealed in the expression of other factors compared with the blank group (*P* > .05). The NC and blank groups had no significant difference. To conclude, the osteoclastic bone absorption was suppressed by CST5‐mediated inhibition of the NF‐κB pathway.

## 4 DISCUSSION

**Figure 8:**
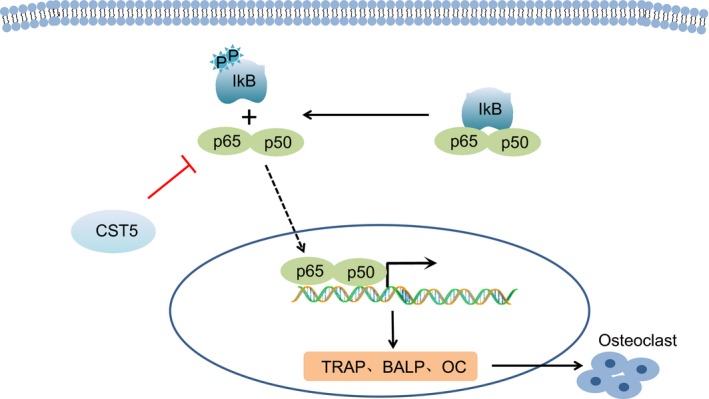
CST5 could inhibit the progression of OP by inhibiting the NF‐κB pathway. CST5 reduced the expression of TRAP, BALP and OC through inhibition of the NF‐κB pathway by which mechanism CST5 conferred inhibitory effect on activation and bone absorption of osteoclasts

Osteoporosis is commonly happened in the older and characterized by damaged bone strength which increases fracture incidence.^24^ Additionally, the rate of bone resorption exceeds the rate of bone formation of osteoclasts largely contributes to OP in postmenopausal women.^25^ The NF‐κB pathway could mediate osteoblast differentiation, and inhibition of the NF‐κB pathway promoted osteoblast differentiation and suppressed osteoclastogenesis.^22^ In the present study, we investigated the effect of CST5 on the activation and bone resorption of osteoclasts through regulation of the NF‐κB pathway, thus affecting the progression of OP. On the whole, our findings suggested that up‐regulation of CST5 could suppress the activation and bone resorption of osteoclasts by inhibiting the NF‐κB pathway in OP (Figure 8).

Initially, the microarray analysis revealed that CST5 was lowly expressed in OP, based on which we speculated that low expression of CST5 may affect OP. In the present study, we found that up‐regulation of CST5 could inhibit the serum levels of TRAP, BALP and OC of rats with OP yet promote the expression of CD61 on the surface of osteoclasts. The role of CST5 was previously revealed in colorectal cancer, in which loss of CST5 promoted tumour differentiation while induction of CST5 exerted a tumour‐suppressive effect on colorectal cancer.^11^, ^26^ More importantly, as a cysteine proteinase inhibitor, CST5 could restrain osteoclast formation through interference of late‐staged differentiation of pre‐osteoclasts.^21^ TRAP was known to be an osteoclast marker, and the increased expression of TRAP was exhibited in osteoblasts and osteocytes in experimental OP rats.^27^ Elevated expression of alkaline phosphatase (ALP) derived from bones and liver was closely associated with bone metabolic marker BAP, and elevation of ALP resulted from bone turnover in postmenopausal osteoporotic women.^28^ OC was considered to be a modulator of bone mineralization, activity of osteoblasts and osteoclasts, and vertebral fracture became susceptible due to increased OC expression.^29^ During the process of bone resorption, CD61 adhesion to bone matrix was utilized by osteoclasts, and lack of CD61 in Glanzmann thrombasthenia osteoclasts exhibited decreased bone resorption.^30^ In the present study, the results showed that the levels of TRAP, BALP and OC in serum were inhibited by up‐regulation of CST5, which indicated that up‐regulation of CST5 may suppress the progression of OP.

From the results of our experiment, we found that the mRNA expression and protein level of RANK, OPG, p65 and IKB were inhibited by up‐regulation of CST5, indicating that overexpression of CST5 could inhibit the NF‐κB pathway. Moreover, osteoclast formation, growth in the number and area of osteoclastic resorption pits as well as bone absorption of osteoclasts were suppressed by inhibition of the NF‐κB pathway mediated by CST5. Bone homeostasis was maintained by bone‐resorbing osteoclasts and bone‐forming osteoblasts.^31^ RANKL has been revealed to be associated with osteoporosis in postmenopausal women.^32^, ^33^ An experiment had stated that RANKL can stimulate osteoclast formation.^34^ Another study has illustrated that osteoclastogenesis, bone resorption and osteoclast activities are inhibited through suppression of RANKL pathway.^35^ RANK is located in the cell membrane of osteoclasts and facilitates osteoclast activation and differentiation.^3^ RANKL was declared to accelerate bone resorption and depression of NF‐κB pathway represses osteoclast differentiation.^36^, ^37^ All the above demonstrations provided evidence that the NF‐κB pathway plays an important role in OP progression and makes simulative effects on bone resorption and activation of OP. In the present study, we found that the NF‐κB pathway could be inhibited by CST5 up‐regulation and by which mechanism the progression of OP was impeded.

Collectively, our study illustrates that CST5 up‐regulation suppresses the activation and bone resorption of osteoclasts by repressing the activation of the NF‐κB pathway, thus further impeding OP progression. Moreover, we confirm that CST5 outlines a potential molecular target for OP treatment. However, further studies are still required to figure out the potential of CST5 in a complex cascade of molecular and cellular network alterations for the treatment of OP.

## CONFLICT OF INTEREST

The authors declare that they have no competing interests.

## AUTHOR CONTRIBUTIONS

FW and CZZ designed the study. FW and WG collated the data, carried out data analyses and produced the initial draft of the manuscript. CZZ and GQZ contributed to drafting the manuscript. All authors have read and approved the final submitted manuscript.
